# Evolution of Robustness to Protein Mistranslation by Accelerated Protein Turnover

**DOI:** 10.1371/journal.pbio.1002291

**Published:** 2015-11-06

**Authors:** Dorottya Kalapis, Ana R. Bezerra, Zoltán Farkas, Peter Horvath, Zoltán Bódi, Andreea Daraba, Béla Szamecz, Ivo Gut, Mónica Bayes, Manuel A. S. Santos, Csaba Pál

**Affiliations:** 1 Synthetic and Systems Biology Unit, Institute of Biochemistry, Biological Research Centre of the Hungarian Academy of Sciences, Szeged, Hungary; 2 Institute for Biomedicine—iBiMED, Health Sciences, University of Aveiro, Aveiro, Portugal; 3 Centro Nacional de Análises Genómico, Parc Científic de Barcelona, Barcelona, Spain; Institute of Science and Technology Austria (IST Austria), AUSTRIA

## Abstract

Translational errors occur at high rates, and they influence organism viability and the onset of genetic diseases. To investigate how organisms mitigate the deleterious effects of protein synthesis errors during evolution, a mutant yeast strain was engineered to translate a codon ambiguously (mistranslation). It thereby overloads the protein quality-control pathways and disrupts cellular protein homeostasis. This strain was used to study the capacity of the yeast genome to compensate the deleterious effects of protein mistranslation. Laboratory evolutionary experiments revealed that fitness loss due to mistranslation can rapidly be mitigated. Genomic analysis demonstrated that adaptation was primarily mediated by large-scale chromosomal duplication and deletion events, suggesting that errors during protein synthesis promote the evolution of genome architecture. By altering the dosages of numerous, functionally related proteins simultaneously, these genetic changes introduced large phenotypic leaps that enabled rapid adaptation to mistranslation. Evolution increased the level of tolerance to mistranslation through acceleration of ubiquitin-proteasome–mediated protein degradation and protein synthesis. As a consequence of rapid elimination of erroneous protein products, evolution reduced the extent of toxic protein aggregation in mistranslating cells. However, there was a strong evolutionary trade-off between adaptation to mistranslation and survival upon starvation: the evolved lines showed fitness defects and impaired capacity to degrade mature ribosomes upon nutrient limitation. Moreover, as a response to an enhanced energy demand of accelerated protein turnover, the evolved lines exhibited increased glucose uptake by selective duplication of hexose transporter genes. We conclude that adjustment of proteome homeostasis to mistranslation evolves rapidly, but this adaptation has several side effects on cellular physiology. Our work also indicates that translational fidelity and the ubiquitin-proteasome system are functionally linked to each other and may, therefore, co-evolve in nature.

## Abstract 2

Tolerance to errors during protein synthesis evolves rapidly through acceleration of protein turnover—a process determined by the combined rates of protein synthesis and degradation. However, this adaptation has deleterious side effects due to its energy costs.

## Author Summary

Although fidelity of information transfer has a substantial impact on cellular survival, many steps in protein production are strikingly error-prone. Such errors during protein synthesis can have a substantial influence on viability and the onset of genetic diseases. These considerations raise the question as to how organisms can tolerate errors during protein synthesis. In this paper, for the first time, we study organisms’ capacity to evolve robustness against mistranslation and explore the underlying cellular mechanisms. A mutant yeast strain was engineered to translate a codon ambiguously (mistranslation). This thereby overloads the protein quality-control pathways and disrupts cellular protein homeostasis. This strain was used to study the capacity of the yeast genome to compensate for the deleterious effects of protein mistranslation. We found that mistranslation led to rapid evolution of genomic rearrangements, including chromosomal duplications and deletions. By altering the dosages of numerous, functionally related proteins simultaneously, these genetic changes introduce large phenotypic leaps that enable adaptation to mistranslation. Robustness against mistranslation during laboratory evolution was achieved through acceleration of protein turnover—a process that was determined by the combined rates of protein synthesis and ubiquitin-proteasome system-mediated degradation. However, as both translation and active degradation of proteins are exceptionally energy-consuming cellular processes, accelerated proteome turnover has substantial energy costs.

## Introduction

Fidelity of protein synthesis has a substantial impact on cellular survival [1,2]. There might be incorporation of one amino acid for another (missense errors), premature termination of protein synthesis, frameshift errors and read-through of stop codons (nonsense errors). Even if the cell has the right protein sequence at the right time, errors can occur during folding, synthesis of posttranslational modifications, or translocation of proteins across membranes. Recent works indicate that many steps in protein production are strikingly error-prone. For example, the average missense error rate during translation is between 10^−3^ and 10^−4^ [3]. While such estimates are rough and are based on a variety of methodologies, they clearly indicate that error rates during protein production are three to five orders of magnitude higher than DNA-replication errors. Perfectly replicated unicellular genomes are commonplace, but perfectly synthesized proteomes never occur. Although the exact amount is currently debated [4,5], it appears that a large fraction of the proteins in eukaryotic cells is defective. Most of these faulty proteins are degraded by the proteasome or aggregate [1]. Indeed, disruption of translational fidelity with aminoglycoside antibiotics kills bacteria [6], cells with impaired proofreading have abnormal morphologies [7,8], and enhanced translational error rates in mammals cause disease [9]. For example, a single mutation in a tRNA synthetase yields widespread translation errors and protein misfolding and consequent death of Purkinje cells in the mouse brain [9]. Moreover, decreased accuracy of protein synthesis due to altered codon–anticodon interactions leads to protein aggregation and saturation of the protein quality-control networks [10]. Similarly, mutants conferring a hyper-accurate translation phenotype grow more slowly than wild-type cells [8,11] as excessively accurate ribosomes are kinetically less efficient than wild-type ribosomes. Thus, accuracy of translation has an optimal value reflecting a trade-off between costs of kinetic proofreading and cellular side consequences of erroneous protein accumulation.

A variety of quality-control mechanisms exist that reduce the rate at which errors occur (error prevention) or limit harmful effects if an error has already been made (error mitigation) [12]. Error reduction may be achieved by an improved proofreading machinery or usage of codons with corresponding highly abundant tRNAs [12]. Furthermore, as protein synthesis errors frequently lead to protein misfolding and aggregation, cells may achieve enhanced tolerance to errors through modification of the cellular chaperone networks, protein degradation pathways, or by the evolution of robust protein folding mechanisms [13–15].

The relative contribution of these pathways to safeguarding the integrity of biological information has remained largely a terra incognita, not least due to the shortage of laboratory evolution studies. To investigate this problem systematically, we took advantage of the availability of a previously engineered *Saccharomyces cerevisiae* strain that mistranslates proteins constitutively at high level [16]. The engineered construct (tRNA_CAG_^Ser^) misincorporates serine (Ser) at leucine (Leu) sites encoded by the CUG codon. As CUG codons in the yeast genome are distributed over 89% of its protein coding genes, this system is ideal to study the impact of protein mistranslation events on a proteome scale. Since more than 60% of the CUG encoded residues are buried in *S*. *cerevisiae* proteins [17], and serine and leucine are chemically very different from each other, mistranslation events generate protein misfolding and proteotoxic stress [10]. To shed light on the evolution of safeguarding mechanisms, we initiated laboratory evolutionary experiments with a strain with initially high mistranslation rate. Fitness rapidly increased during the course of laboratory evolution and was mediated by large-scale chromosomal duplication and deletion events. Robustness to translational errors was achieved through accelerated protein turnover, albeit at a considerable energetic cost.

## Results

### Mistranslation Has a Substantial Fitness Cost

**Fig 1:**
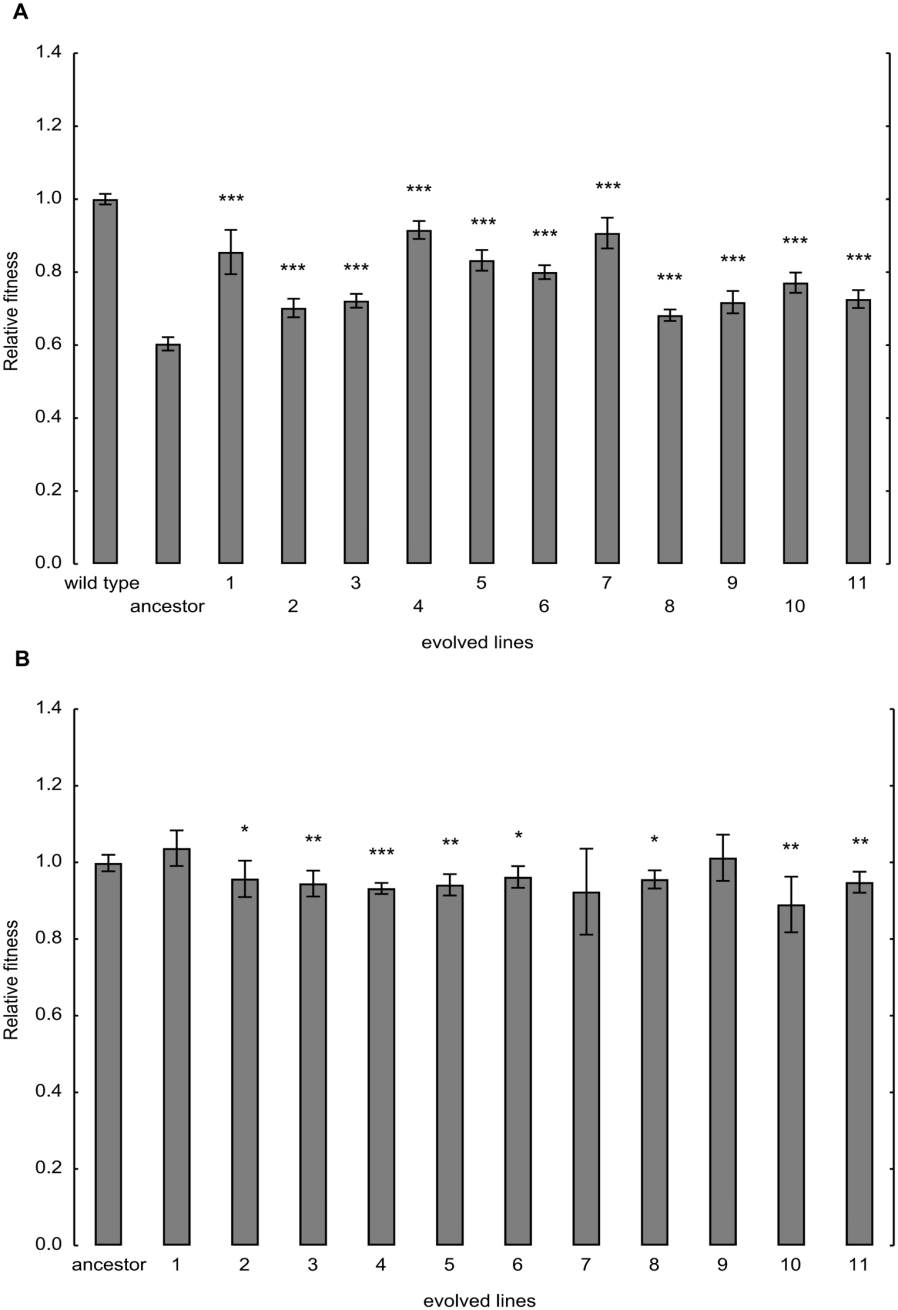
Fitness changes during laboratory evolution. (A) Fitness of the ancestor and the evolved lines under high mistranslation rate. The ancestor and evolved lines carry the mistranslation-causing tRNA_CAG_^Ser^ construct. The ancestor is isogenic to the wild type, with the only exception being that the latter carries an empty vector instead of tRNA_CAG_^Ser^. Growth rate (calculated by monitoring optical density) was used as a proxy for fitness. Fitness values were normalized to the wild-type control. The bars indicate mean ± 95% confidence interval. Mann-Whitney U test was used to assess difference in fitness between ancestor and evolved lines. *** indicates *p* < 0.001. (B) Fitness of the ancestor and evolved lines under low mistranslation rate. tRNA_CAG_^Ser^ was swapped for the corresponding empty vector in the ancestor and the evolved lines. Growth rate (calculated by monitoring optical density) was used as a proxy for fitness. Fitness values were normalized to the wild-type control carrying no tRNA_CAG_^Ser^ construct. The bars indicate mean ± 95% confidence interval. Mann-Whitney U test was used to assess difference in fitness between ancestor and evolved lines. */**/*** indicates *p* < 0.05/0.01/0.001, respectively.

Using a synthetic tRNA_CAG_^Ser^ construct on a plasmid, we have previously engineered codon misreading in a diploid yeast strain that misincorporates serine at the leucine CUG codon [16,18]. A previous quantitative mass-spectrometry study indicates that the tRNA_CAG_^Ser^ misincorporates 1.4% of serine at the CUG sites [19]. As the background mistranslation error in yeast is around 0.001% [20], the construct generates a 1,400-fold increase in mistranslation at CUG sites on a proteome-wide scale. We used established protocols specifically designed to measure fitness in yeast populations [21,22]. Growth was assayed by monitoring the optical density at 600 nm wavelength (OD_600_) of liquid cultures of each strain on synthetic complete (SC) leucine dropout medium. The tRNA_CAG_^Ser^ construct reduced growth rate by 40%, suggesting that mistranslation has a substantial fitness cost in an otherwise stress-free environmental condition (Fig 1A).

### Fitness Loss due to Mistranslation Is Rapidly Mitigated during Evolution

Next, we initiated laboratory evolutionary experiments, starting from a single clone, that carried the tRNA_CAG_^Ser^ plasmid. Eleven replicate populations were cultivated in a standard laboratory medium and 1% of each parallel evolving culture was diluted into a fresh medium every 48 h. Populations were propagated for approximately 250 generations (70 d). To control for potential adaptation to the medium, we also established 11 populations starting from the isogenic wild-type strain that carried the empty vector (instead of tRNA_CAG_^Ser^). Next, we isolated a single colony from each independently evolved population after 70 d of evolution. All starting and evolved lines were subjected to high-throughput fitness measurements by monitoring growth rates in liquid cultures. We found that the evolving wild-type control lines showed a marginal 3.7% fitness improvement (S1 Fig). By contrast, the fitness of the lines with tRNA_CAG_^Ser^ improved by 13.3%–51.7%, and many of them approximated wild-type fitness (Fig 1A).

Plasmid sequence analysis in the evolved lines confirmed that the rapid fitness gain was not due to defective mutations in tRNA_CAG_^Ser^ (S2 Fig). Next, tRNA_CAG_^Ser^ was swapped for the corresponding empty vector in all starting and evolved lines. Most evolved lines showed 3%–11% growth deficit compared to their ancestor (Fig 1B). Thus, the mutations that have accumulated during the course of laboratory evolution are favorable when mistranslation rate is high but generally reduce fitness otherwise.

### Translation and the Ubiquitin-Proteasome System Are Two Main Targets of Selection

To investigate the molecular changes underlying adaptive evolution in the laboratory, we re-sequenced the complete genomes of 11 independently evolved clones, all of which showed substantial fitness improvements under high mistranslation rate. In total, we identified 431 independent mutational events (SNPs and large genomic rearrangements). On average, we detected 39.18 point mutations, 1 deletion, and 2 duplications per line. A strong pattern of parallel evolution emerged at the level of genes and functional modules. Of the mutated genes, 26% with non-synonymous mutations were shared by at least two lines, and some were shared extensively (S1 Table). Several functional units were repeatedly mutated. The examples include rRNA maturation (*MRD1* [S000006316]), transcription initiation and elongation (*BDF1* [S000004391], *TFG2* [S000003237]), and the ubiquitin-proteasome system (*SSM4* [S000001292], *HSM3* [S000000476]).

Large-scale duplications (including segmental or whole-chromosome duplication) and genomic deletions were also prevalent (Table 1). In total, 33 large-scale duplication and partial chromosome deletion events were observed. Many of the evolved lines accumulated 4–5 gross chromosomal rearrangements. Strikingly, however, only seven major different types of such events were observed, most of which occurred repeatedly. For example, over 90% of the evolved lines lost a small part of one copy of chromosome V, which spanned over 127 kb. The evolved lines also frequently carried duplications of large segments of chromosomes IV (540 kb) and VII (238 kb). Borders of these duplicated genomic regions were flanked by transposon of yeast (Ty) elements, indicating that the observed genomic changes were mediated by homologous recombination events between Ty elements (S2 Table).

**Table 1:** Summary of the detected chromosomal duplications and deletion events in the evolved lines.

Chromosome	**Region**	**Aneuploidy**	**GO**	**Evolved lines**										
				1	2	3	4	5	6	7	8	9	10	11
chrIII	whole	trisomy	cell redox homeostasis								●			
			regulation of cellular amino acid metabolic process								●			
			ribosomal small subunit assembly								●			
chrIV	992 kb to end	trisomy	substrate-specific transmembrane transporter activity		●	●	●	●	●		●	●	●	●
chrV	450 kb to end	monosomy	ubiquitin-specific protease activity	●*	●	●	●	●	●		●	●	●	●
chrVII	574 kb to 812 kb	trisomy	proteasome core complex assembly	●										
			response to reactive oxygen species	●										
			RNA polymerase II transcription elongation factor activity	●										
chrIX	whole	trisomy	negative regulation of gluconeogenesis	●					●		●			
			ribosomal small subunit export from nucleus	●					●		●			
			termination of RNA polymerase II transcription	●					●		●			
chrXII	whole	trisomy	ribosome biogenesis	●	●	●	●			●	●	●		●
			ribosomal large subunit assembly	●	●	●	●			●	●	●		●
chrXVI	810 kb to 844 kb	monosomy	none											●

The affected chromosomes, the extent, the direction of the changes, and biological processes that are enriched significantly (*p* < 0.05) in the regions are indicated. Functional enrichment analysis was done using FunSpec, a web-based cluster interpreter for yeast [23].

* indicates partial monosomy of chrV from 450 kb to 498 kb.

The repeated evolution of chromosomal rearrangements at the same breakpoint suggests that these mutational events confer adaptive advantage to the carrying cells. To investigate this issue further, we grouped the mutated genes into several major functional categories based on database information. The observed large-scale duplication events were enriched in genes involved in ribosomal biogenesis (e.g., *DRS1* [S000003931], *SOF1* [S000003934], *RIX7* [S000003957]), rRNA processing (e.g., *GRC3* [S000003958], *NOC3* [S000003992], *SDO1* [S000004012]), and proteasome core complex assembly (*PRE9* [S000003367]). Significantly, the small genomic region repeatedly deleted on chromosome V carries two genes encoding deubiquitinating enzymes (*UBP3* [S000000953], *UBP5* [S000000946]).

**Fig 2:**
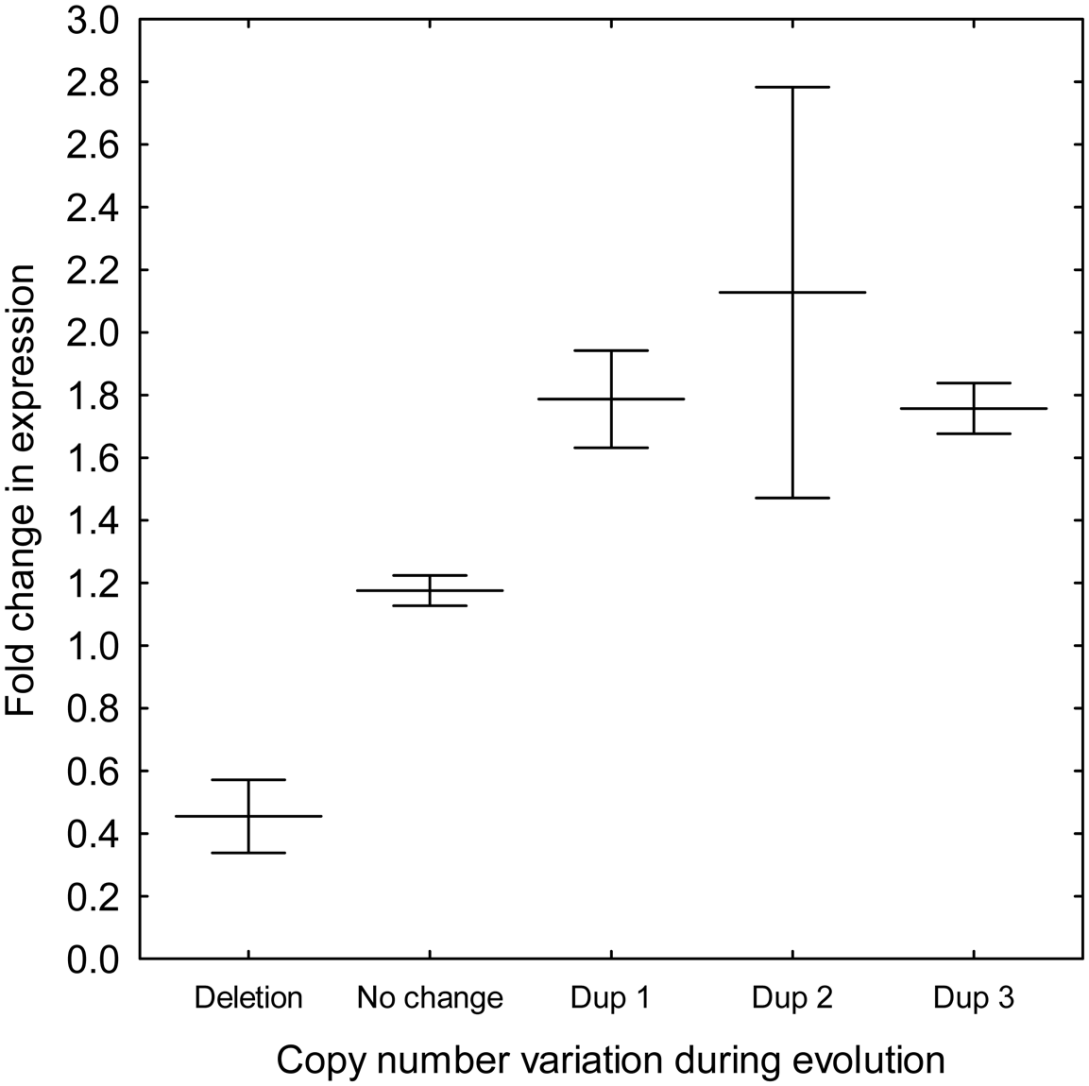
Gene expression changes as a function of copy number variation. The figure shows mRNA expression fold change in evolved line 1 relative to the corresponding ancestor. As expected, loss of a small part of one copy of chromosome V (deletion) leads to reduced mRNA levels of the corresponding genes, while the opposite is true for genes carried on duplicated segments of VII (Dup1), IX (Dup2) and XIII (Dup3). Center lines show mean ± 95% confidence interval. Fold change differences across the three main categories (duplication, deletion, and no change) are highly significant (*t* test, *p* < 10^−6^ for each comparison).

Last, we studied the impact of the accumulated mutations on genomic expression. The analysis focused on two independently evolved strains (lines 1 and 4), both of which carried typical large-scale genomic rearrangements (Table 1). We grew these strains to mid-log phase in standard laboratory medium and measured genomic expression relative to the ancestor strain (DNA microarrays were used for this purpose). Only genes that showed at least a 2-fold change in expression were considered further. As expected, copy number variation had a significant impact on gene expression in the evolved lines (Fig 2, see S3 Table for the full dataset).

To reveal the functional enrichment of genes with changed expression unrelated to chromosomal duplication or deletion, we focused on genomic segments that have not undergone copy number changes during the course of evolution. The two strains showed correlated changes in genomic expression (Spearman correlation, r = 0.54 *p* < 10^−6^). Given this correlation, we focused on the set of genes with expression changes in the same direction in the two strains. A total of 425 genes were affected in both strains, of which 168 were induced and 257 repressed, relative to the ancestor. Functional enrichment analysis revealed that genes involved in rRNA processing, ribosome biogenesis and amino acid biosynthesis were up-regulated (Table 2).

**Table 2:** Functional enrichment of genes showing altered transcription in the evolved lines relative to the ancestor.

**Line**	**Direction of change**	**Gene ontology (GO)**	**Number of genes affected**
Evolved line 1	up-regulation	ribosome biogenesis	67
		rRNA processing	62
		ribosomal large subunit assembly	18
	down-regulation	electron transport chain	17
		glycogen biosynthetic process	7
Evolved line 4	up-regulation	ribosome biogenesis	25
		rRNA processing	21
		cellular amino acid biosynthetic process	11
		ribosomal large subunit assembly	6
	down-regulation	cellular response to oxidative stress	22
		glycogen biosynthetic process	7

Analysis was done using FunSpec, a web-based cluster interpreter for yeast [23].

The precise molecular mechanisms underlying up-regulation of ribosomal genes in the evolved strains are unknown. It is worth noting, however, that genes encoding ribosomal proteins form the tightest cluster of coordinately regulated genes, and one of the most prominent transcription factors controlling the ribosomal regulon is *IFH1* (S000004213) [24]. Significantly, *IFH1* is located on chromosome XII, and this chromosome has undergone a complete duplication during the course of laboratory evolution (Table 1).

Taken together, the genomic analyses demonstrate that evolution preferentially targeted genes involved in translation and the ubiquitin-proteasome system.

### Increase in Protein Translation Rate during Evolution

**Fig 3:**
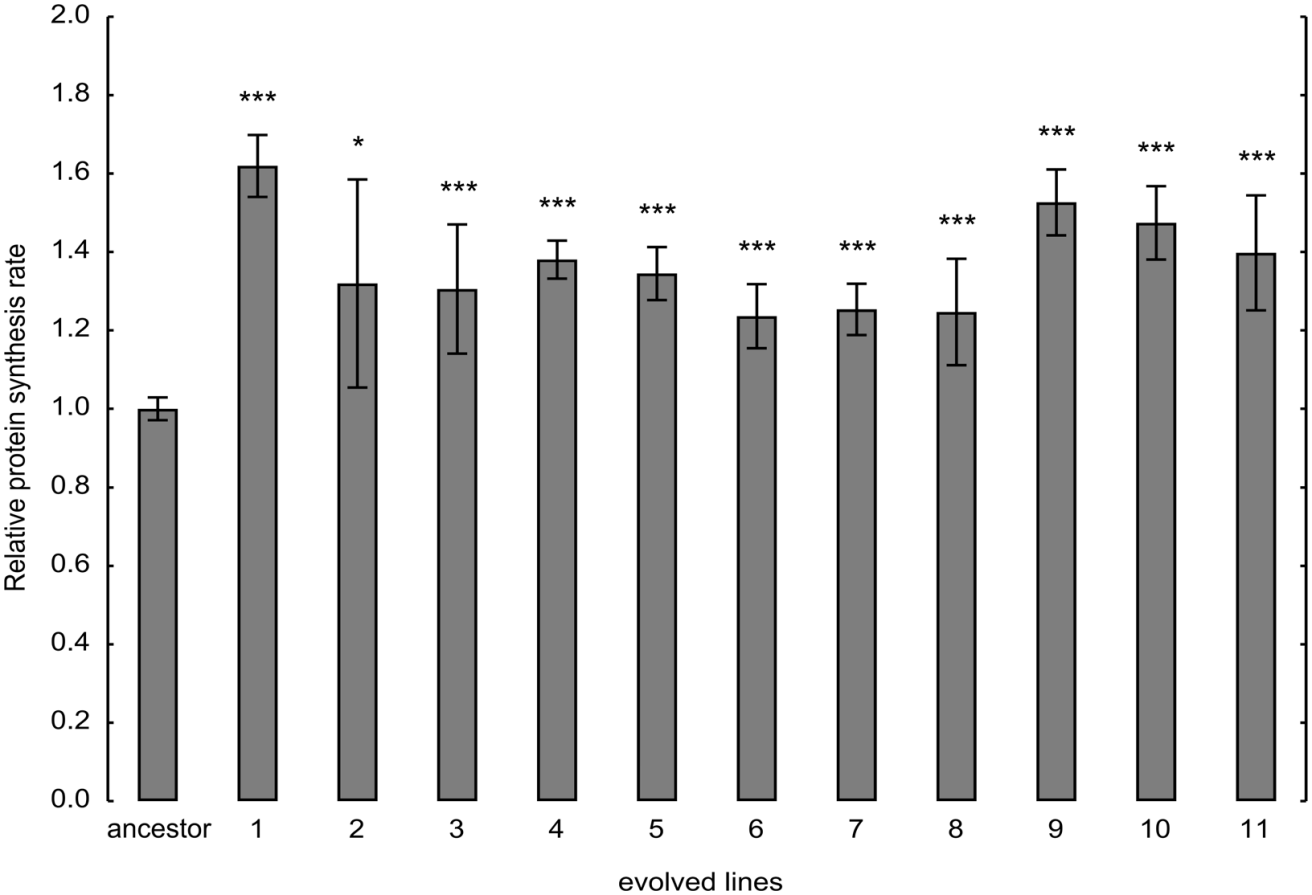
Evolution of protein synthesis rate. Amino acid incorporation rate was measured by protein pulse labeling with [^14^C(U)]-L-Amino Acid mixture. The bars indicate mean ± 95% confidence interval. Two-sample *t* test was used to assess difference in protein synthesis rate between ancestor and evolved lines. */**/*** indicates *p*-value < 0.05/0.01/0.001.

Based on results of the previous section, we next asked whether the observed mutations influence protein synthesis rate. We used an established biochemical assay; protein pulse labeling with [^14^C(U)]-L-Amino Acid Mixture [25,26]. To ensure that changes in protein synthesis rate reflect the impact of the accumulated mutations in the evolved lines (rather than growth rate mediated effects of mistranslation), tRNA_CAG_^Ser^ was swapped for the corresponding empty vector in the ancestor and the evolved lines. Ancestor and evolved cells were incubated with a mixture of carbon-14 labeled L-amino acids. The rate of carbon-14 labeled amino acids incorporation in newly synthesized proteins reflects directly the rate of mRNA translation in vitro. Protein synthesis was quantified according to carbon-14 labeled L-amino acids incorporation detected by a scintillation counter. We found that the evolved lines show higher protein synthesis rate than the corresponding ancestor (Fig 3).

### Reduction of Mistranslation Rate during Evolution

**Fig 4:**
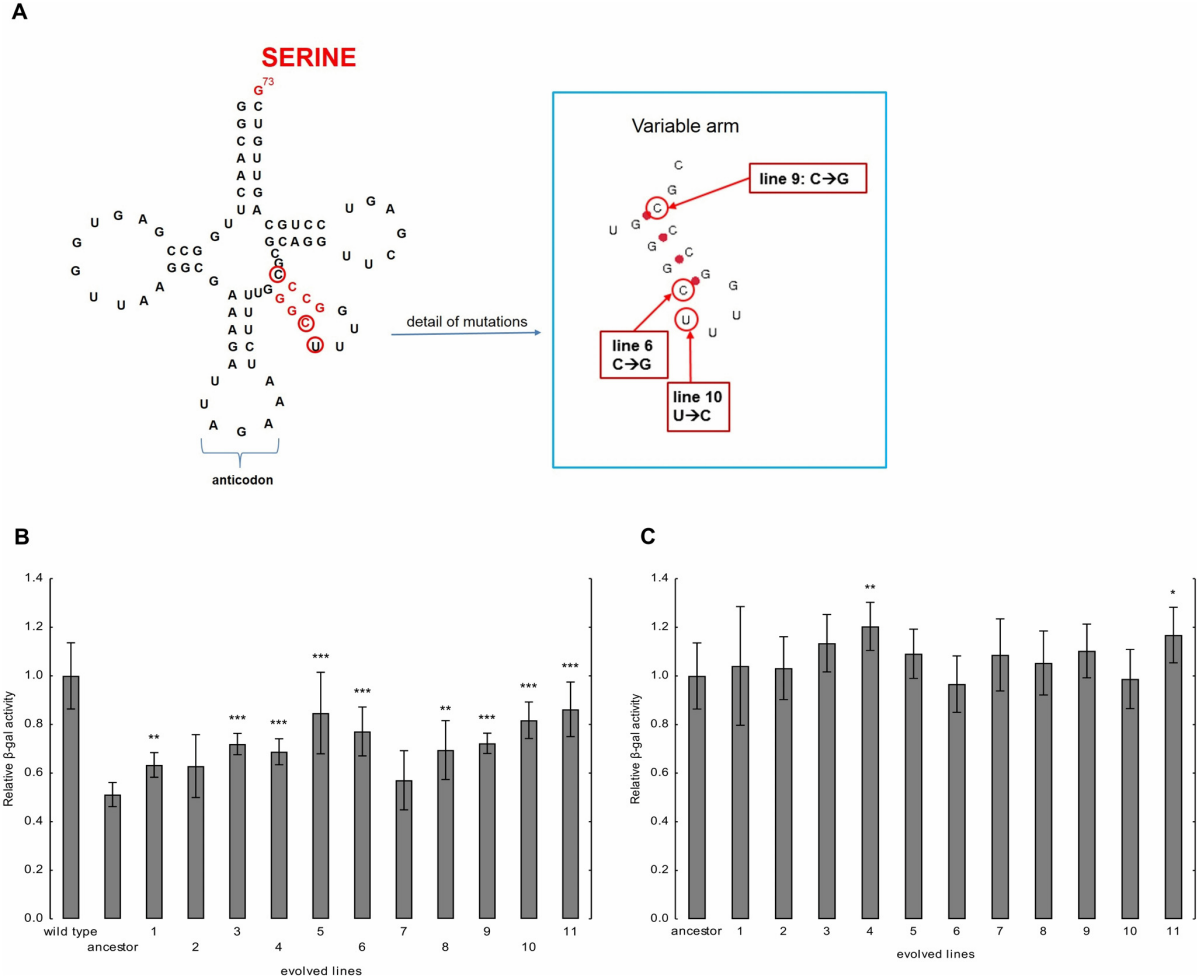
Evolution of mistranslation rate. (A) Mutations in the variable arm of the tS(AGA)D3 serine tRNAs. The variable arm mutated in three lines independently (lines 6, 9, and 10). The identity elements for the SerRS are indicated in red (discriminator base G73 and the GC base pairs in the variable arm). Structure of the molecule was predicted by tRNAscan-SE analysis. (B) Evolution of mistranslation rate. The figure shows β-galactosidase enzyme activities in the ancestor and evolved lines, all of which carry the mistranslation causing tRNA_CAG_^Ser^ construct. The ancestor is isogenic to the wild type, with the only exception being that the latter carries an empty vector instead of tRNA_CAG_^Ser^. Enzyme activities were normalized to the enzyme activity measured in wild-type control carrying no tRNA_CAG_^Ser^ by normalization to the total amount of β-galactosidase protein (quantified by western blot). The bars indicate mean ± 95% confidence interval. Mann-Whitney U test was used to assess difference in fitness between ancestor and evolved lines. **/*** indicates *p* < 0.01/0.001, respectively. (C) β-galactosidase enzyme activities in the ancestor and the evolved lines carrying no tRNA_CAG_^Ser^. Enzyme activities were normalized to the enzyme activity measured in the wild type after normalization to the total amount of β-galactosidase protein quantified using western blot. The bars indicate mean ± 95% confidence interval. Mann-Whitney U test was used to assess difference in fitness between ancestor and evolved lines */** indicates *p* < 0.05/0.01, respectively.

Several mutated genes were involved in the regulation of tRNA transcription (RNA polymerase III), tRNA export (*SOL1* [S000005317]), tRNA surveillance and degradation (*TRF5* [S000005243]). A serine tRNA was also repeatedly mutated, mostly in the variable arm of this molecule (Fig 4A, S4 Table), which is recognized by the seryl-tRNA synthetase (SerRS). Indeed, the yeast SerRS recognizes the three G-C base-pairs of the variable arm of serine tRNAs and the discriminator base at position 73 (G73). Based on these observations, we assumed that evolution has acted to alter tRNA stability and cellular abundance. Changes in the tRNA pool could subsequently reduce the rate of mistranslation during the evolution period.

Misreading activity of tRNA_CAG_^Ser^ was then tested by using a previously developed β-galactosidase assay [10]. Briefly, the *Escherichia coli LacZ* gene contains 54 CTG codons, and misincorporation of serine at these leucine codons generates a combinatorial array of mutant β-galactosidase molecules. The altered stability of these statistical protein ensembles can be quantified using thermal denaturation assays [10]. The high number of CTG codons present in the *LacZ* gene combined with the different chemical properties of serine (polar amino acid) and leucine (hydrophobic amino acid) make β-galactosidase a highly sensitive reporter, allowing for monitoring misreading activity by tRNA_CAG_^Ser^. Prior to evolution, presence of tRNA_CAG_^Ser^ caused a 62% reduction in β-galactosidase activity (Fig 4B). Next, we compared β-galactosidase activities of the ancestor and the evolved tRNA_CAG_^Ser^ carrying lines. A relatively small but significant increase in β-galactosidase activity was found in the evolved lines, indicative of a slight reduction of misreading by tRNA_CAG_^Ser^. When tRNA_CAG_^Ser^ was swapped for the corresponding empty vector, all evolved lines had high β-galactosidase activities, similar to that of the ancestor (Fig 4C). This result confirms that the presence of tRNA_CAG_^Ser^ is crucial for serine misincorporation.

Taken together, these results indicate that evolution acted to reduce mistranslation rate, probably by influencing tRNA aminoacylation, tRNA pool distribution, or usage during translation. Indeed, we note that quantification of tRNA expression by northern blot revealed a small but significant reduction in the cellular abundance of mistranslating tRNA in three evolved lines (S3 Fig).

### Reduction of Protein Aggregation Propensity during Evolution

Even if a translational error is not prevented, the fitness consequences that ensue could still be reduced [1,12]. Fitness cost of mistranslation could be partly due to protein misfolding, protein aggregation, and consequent induction of cellular toxicity [1]. Indeed, a prior work showed that tRNA_CAG_^Ser^ in yeast initiates a proteotoxic stress response and activates the unfolded protein response pathway [10]. Therefore, we hypothesized that changes in the ubiquitin-proteasome system mitigate the harmful consequences of mistranslation by reducing the extent of protein aggregation.

**Fig 5:**
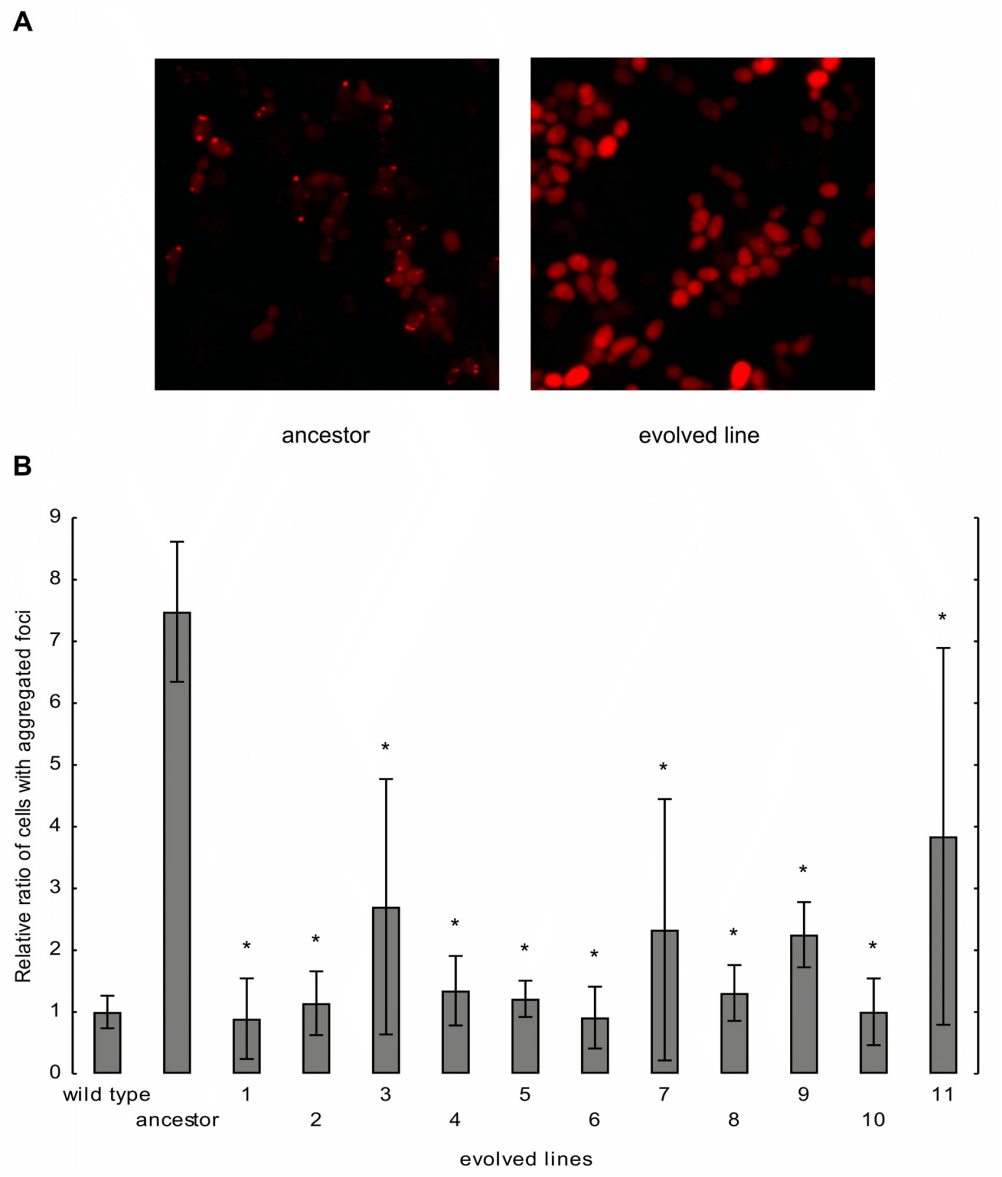
Evolution of protein aggregation rate. (A) Distribution of VHL-mCherry in ancestor and evolved cells. (B) Changes of protein aggregation rate during evolution. The ancestor and evolved lines carry the mistranslation-causing tRNA_CAG_^Ser^ construct. The ancestor is isogenic to the wild type, with the only exception being that the latter carries an empty vector instead of tRNA_CAG_^Ser^. The number of cells with and without aggregated foci was counted, and the ratio of cells with aggregated foci was calculated. This ratio calculated in ancestor and evolved lines was normalized to the ratio calculated in the wild type. The ratio of the cells with aggregated foci can be used as a proxy for protein aggregation rate. The bars indicate mean ± 95% confidence interval. Mann-Whitney U was used to assess difference in fitness between ancestor and evolved lines. * indicates *p* < 0.05.

To shed light on a possible link between mistranslation and protein aggregation, we measured the level of cellular aggregation in cells subjected to mistranslation and compared it with that of the wild-type control. An established method based on aggregation of a fluorescently tagged human protein (VHL, von Hippel–Lindau) was applied [27]. Active quality-control machinery in wild-type yeast prevents the aggregation of VHL [28]. However, upon overload of the quality-control machinery (including Hsp70 and Hsp90 chaperone complexes), misfolded VHL molecules form aggregates that are seen as foci in the cells [27]. As the fluorescent tag (mCherry) remains functional, localization of aggregation spots in the cells are detectable by fluorescence microscopy. We found that tRNA_CAG_^Ser^ initiated aggregation of VHL in the ancestor (Fig 5A). This result demonstrates that targeting of VHL-mCherry for proteasomal degradation is compromised in these cells. In sharp contrast, the evolved strains showed substantially decreased VHL focus formation, indicative of a reduced aggregation propensity in these lineages (Fig 5B).

One may argue that aneuploidy, such as partial chromosomal duplications found in the evolved lines, are expected to have an opposite effect on protein aggregation, since these genomic changes can overload the protein quality-control machinery [28]. Nevertheless, this is no longer so when aneuploidy-tolerating mutations in a deubiquitinating enzyme (such as Ubp3p) are also present [29]. We previously showed evidence for the loss of a single genomic copy of *UBP3* in most of the evolved lines. In addition, we have also demonstrated that partial chromosomal duplications are beneficial for speeding up translation (due to the overrepresentation of ribosome-associated genes in these genomic regions). Therefore, these genomic rearrangements are more likely to be favorable, without any negative effects on aggregation. As a net outcome, we found that the level of protein aggregation is reduced, rather than increased, in the evolved lines (Fig 5B).

### Enhanced Proteasome Activity in the Evolved Lines

Based on the above findings, we next asked whether selection acted to increase proteasome-mediated degradation of misfolded proteins. Indeed, short-lived proteins display higher average aggregation propensity [30], indicating that efficient degradation of high-turnover proteins is maintained to minimize the danger of aggregation. The genomic analysis is consistent with the hypothesis, as genes related to the ubiquitin-proteasome system were repeatedly mutated (S1 Table). For example, three independently evolved lines carried mutations in the *HSM3* gene, which encodes a proteasome-interacting protein involved in the assembly of the 19S proteasomal regulatory particle. Perhaps most significantly, a small segment of chromosome V carrying genes of deubiquitinating enzymes (*UBP3*, *UBP5*) was repeatedly lost in over 90% of the evolved lines (Table 1).

**Fig 6:**
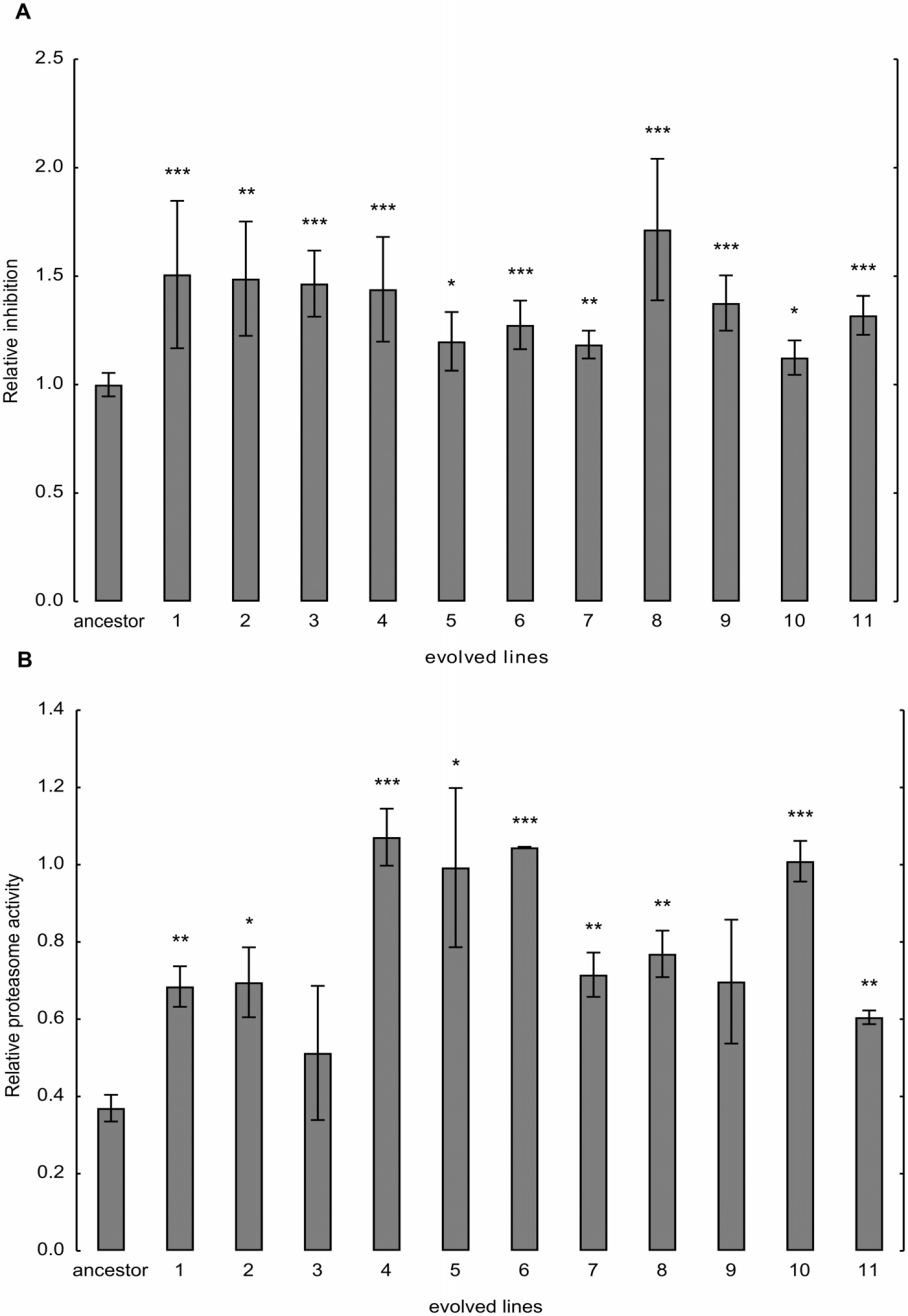
Changes in the ubiquitin-proteasome system during evolution. (A) Inhibition of the ancestor and the evolved lines by cycloheximide. To test the sensitivity of the populations to protein synthesis inhibitor, the growth medium was supplemented with sublethal dosage of cycloheximide (0.06 μg/ml). Inhibition rates were calculated by comparing growth rates in drug-containing and drug-free media, and were normalized to that of the wild-type control. The bars indicate mean ± 95% confidence interval. Mann-Whitney U test was used to assess difference in fitness between ancestor and evolved lines. */**/*** indicates *p*-value < 0.05/0.01/0.001, respectively. To ensure that changes in inhibition rates do not simply reflect the direct effects of mistranslation, tRNA_CAG_^Ser^ was swapped for the corresponding empty vector in the ancestor and the evolved lines. (B) Changes in proteasome activity during evolution. Proteasome activities were normalized to the activity measured in wild-type control carrying no tRNA_CAG_^Ser^. The bars indicate mean ± standard error. Two-sample *t* test was used to assess the difference in proteasome activity between the ancestor and the evolved lines. */**/*** indicates *p*-value < 0.05/0.01/0.001, respectively.

Tagging of a target protein by ubiquitin specifies cellular location and frequently directs it to the 26S proteasome for degradation [31]. Importantly, the ubiquitin-proteasome system is actively involved in eliminating misfolded proteins. Proteasome-associated deubiquitinating enzymes, such as Ubp3p, cleave ubiquitin–protein bonds, and thereby enhance the free intracellular level of ubiquitin [32]. By reversal of ubiquitination, Ubp3p diverts proteins away from the proteasome system. Accordingly, reduced Ubp3p level decreases the intracellular level of free ubiquitin and simultaneously increases the fraction of proteins destined for destruction [32]. Ubiquitin is critical for the survival of yeast cells in the presence of protein synthesis inhibitor cycloheximide [33]. Therefore, the evolved lines are expected to show increased sensitivities to this agent. This was indeed so (Fig 6A).

Along with the observed mutations, a biochemical assay supports the notion that proteasome activity changed during the course of laboratory evolution. Proteasome activity of the ancestor and the evolved lines was quantified as described previously [34], using a fluorogenic peptide as substrate. Briefly, total protein was extracted from exponentially growing cells and activity was determined for 100 μg of protein extract in assay buffer with s-LLVY-MCA. The measured fluorescence emission showed that proteasome chymotrypsin-like activity increased 1.4–2.8-fold in the laboratory evolved lines compared to the ancestor (Fig 6B).

### Evolved Lines Exhibit Increased Glucose Uptake

Both translation- and proteasome-mediated degradation of proteins are exceptionally energy-consuming cellular processes. Therefore, the accelerated proteome turnover is expected to incur substantial energy costs, leading to fitness deficit when external nutrients are limited. Indeed, in agreement with expectations, the evolved lines showed reduced growth rate in glucose- and amino-acid–limited medium compared to their ancestor (S4A and S4B Fig).

**Fig 7:**
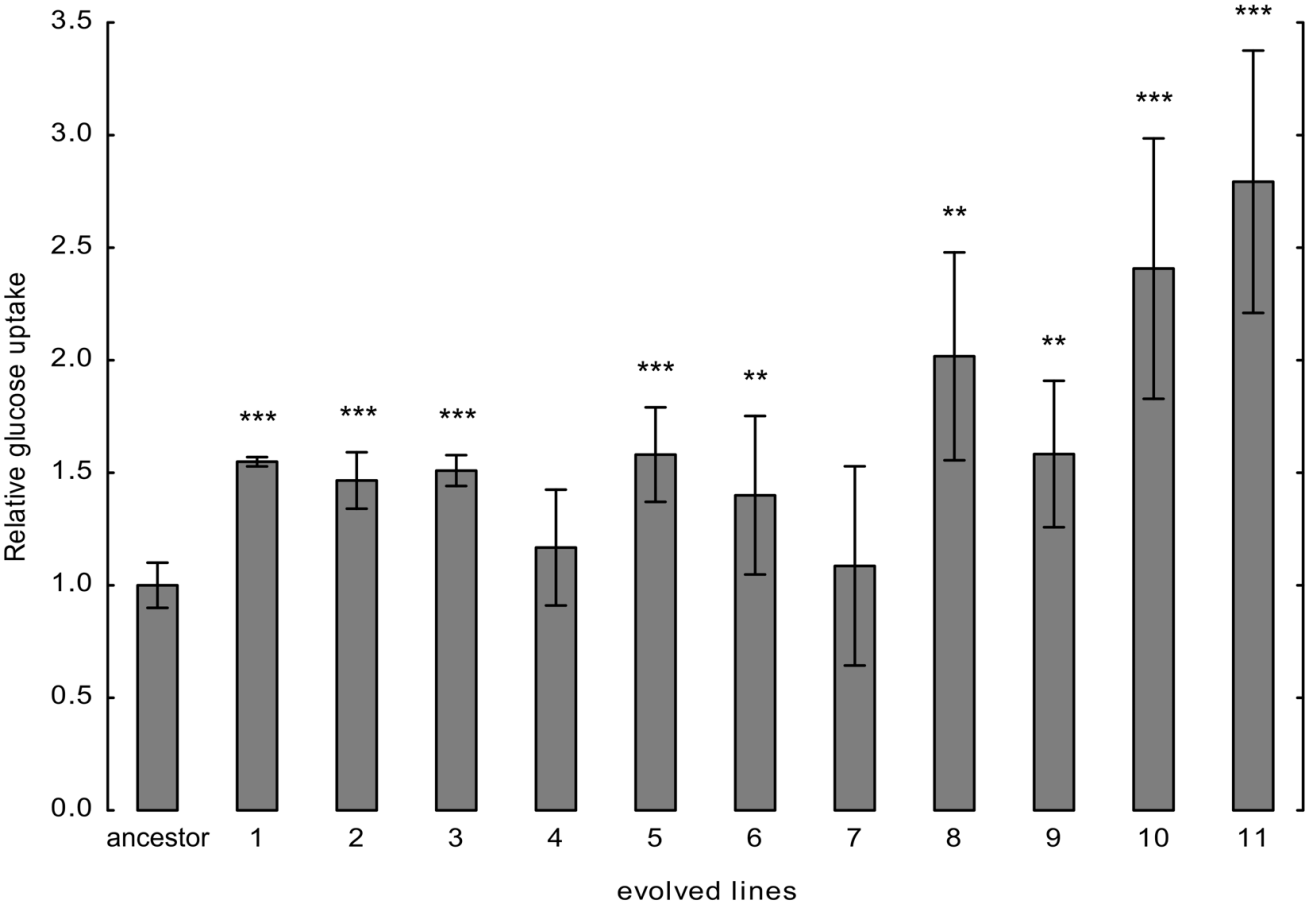
Glucose uptake in the ancestor and the evolved lines. After 15 h of growth, optical densities and medium glucose content were measured in parallel in the ancestor and the evolved lines. Glucose uptake rates were estimated by the drop of glucose content per cell. Glucose uptake rate per cell was normalized to that of the ancestor value. The bars indicate mean ± 95% confidence interval. Two-sample *t* test was used to assess difference in glucose uptake between the ancestor and the evolved lines. */**/*** indicates *p*-value < 0.05/0.01/0.001, respectively. To ensure that changes in glucose uptake reflect the impact of the accumulated mutations in the evolved lines (rather than the direct effects of mistranslation), tRNA_CAG_^Ser^ was swapped for the corresponding empty vector in the ancestor and the evolved lines.

To investigate whether the enhanced energetic costs of accelerated protein turnover and the associated fitness deficit invokes a selection pressure to alter cellular physiology, we measured glucose uptake using established protocols [35]. The comparison revealed that evolved cells generally internalized more glucose molecules than the ancestor cells did (Fig 7).

Several mutations could be responsible for the observed increase in glucose uptake, two of which are especially noteworthy. Nine out of 11 strains carried a segmental duplication of chromosome 4, which spans over 540 kb (Table 1). This small region contains three genes involved in hexose transport (*HXT3* [S000002753], *HXT6* [S000002751], and *HXT7* [S000002750]). Significantly, prior work showed that when yeast is evolving in a glucose-limited environment, populations amplify an overlapping region of chromosome IV, which includes these high-affinity hexose transporters [36,37]. Moreover, *MTL1* (S000003255), a membrane sensor of stress during glucose starvation, was also mutated in seven lines independently. Taken together, the genetic and biochemical data suggest that the evolved lines demand more glucose for cellular proliferation.

### Impaired Ribosome Destruction As a Deleterious Side Effect of Adaptation

**Fig 8:**
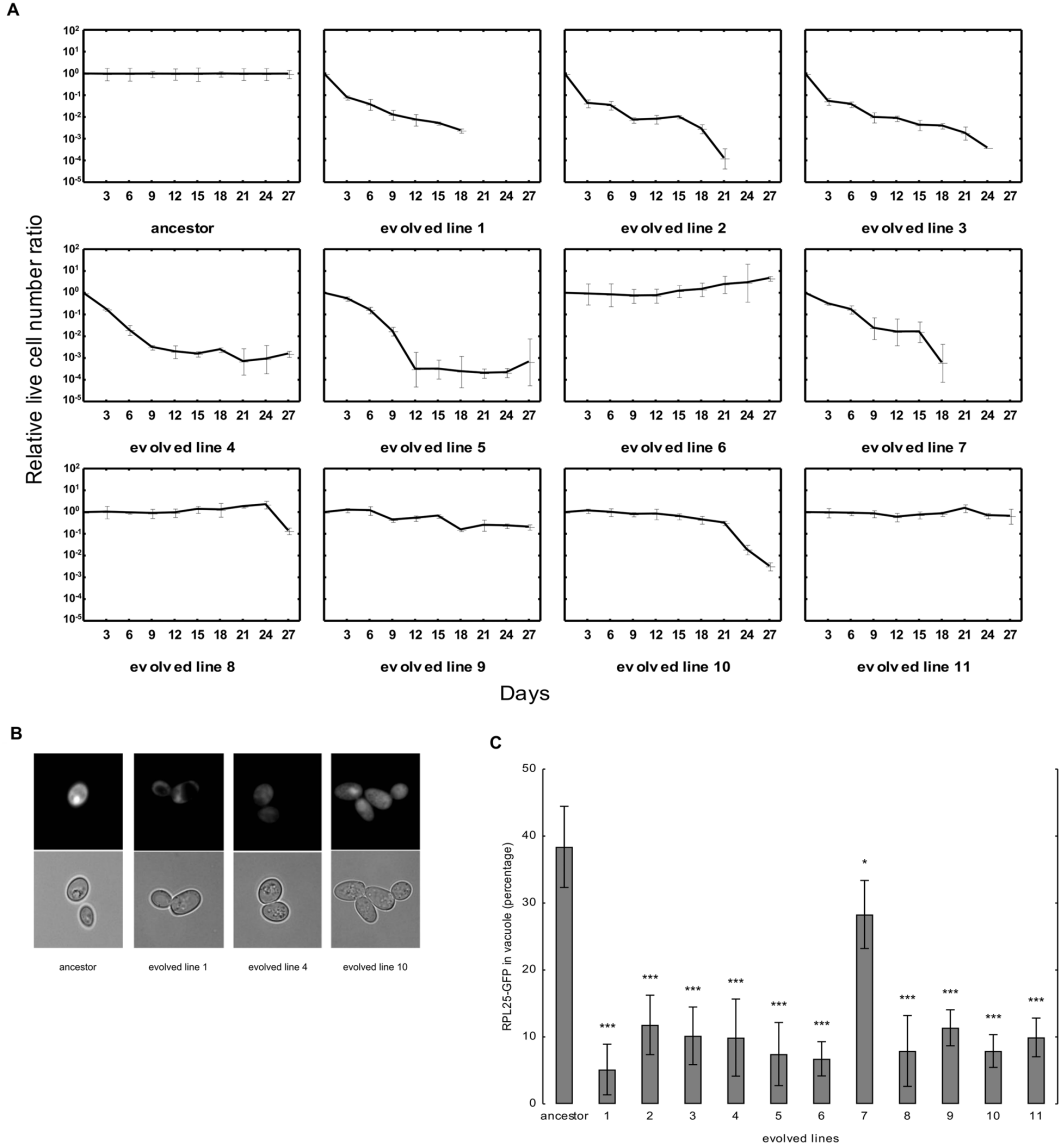
Side effects of adaptation to mistranslation. (A) Survival of the ancestor and the evolved lines upon prolonged starvation. Colony forming units were used as a proxy for live cell numbers. The colony forming units were counted every 3 d, and the ratio of live cells were calculated and normalized to the ancestor (upper left panel). The bars indicate mean ± 95% confidence interval. To ensure that changes in cell survival reflect the impact of the accumulated mutations (rather than the direct effects of mistranslation), tRNA_CAG_^Ser^ was swapped for the corresponding empty vector in the ancestor and in the evolved lines. (B) Distribution of Rpl25p-GFP in ancestor and evolved lines carrying tRNA_CAG_^Ser^. (C) Rpl25p-GFP relocalization upon starvation in the ancestor and the evolved lines carrying tRNA_CAG_^Ser^. Cells were grown to mid-log phase in starvation conditions for 24 h and were analyzed by epifluorescence microscopy. Cells with vacuolar fluorescence were manually counted. At least eight randomly chosen microscopic fields were counted, and approximately 100 cells per line were counted. The bars indicate mean ± 95% confidence interval. Mann-Whitney U test was used to assess the difference in Rpl25p-GFP relocalization between ancestor and evolved lines. *** indicates *p*-value < 0.001.

Last, we tested the response of the evolved lines to complete nutrient depletion. Using standard lifespan assays [38], we found that 8 out of the 11 studied evolved lines rapidly lost viability upon prolonged culturing in starvation (Fig 8A).

We suspected that changes in the ubiquitin-proteasome system could partly be responsible for these changes. Due to the exceptionally high energetic costs of translation, mature ribosomes are selectively degraded by autophagy upon nutrient starvation [39]. This pathway, termed ribophagy [39,40], has an important role in adjusting the number of ribosomes to match the cellular needs. Importantly, ribophagy crucially depends on the expression of *UBP3* [39]. Therefore, the evolved lines with loss of a single genomic copy of *UBP3* may exhibit impaired capacity to degrade mature ribosomes.

To analyze quantitative changes in ribosome destruction, an established protocol was used [27]. The ribosomal protein (Rpl25p [S000005487]) was tagged with green fluorescent protein (GFP). Whereas the fused protein is distributed evenly in the cytoplasm under nutrient-rich conditions, the protein accumulates in the vacuole during starvation [27]. As previously demonstrated, the level of cellular relocalization of this construct is a reliable indicator of ribosome turnover [27]. The evolved lines carried a single genomic copy of the *UBP3* gene, the only exception being line 7, where both copies remained intact (Table 1). Remarkably, all lines with reduced *UBP3* dosage showed markedly reduced Rpl25p–GFP levels in the vacuole during starvation compared to both line 7 and the corresponding ancestor (Fig 8B and 8C). We note also that Rpl25p–GFP level was still somewhat lower in line 7 than that in the ancestor, indicating the existence of other mutations influencing ribophagy.

Although we cannot exclude the possibility that some degradation occurs from unrelated processes, these results clearly indicate that vacuolar processing of Rpl25p–GFP proteins is impaired in the evolved lines. Finally, mortality rate upon starvation is expected to be a complex process also dependent on the rate of using the energy resources of the cell. Future works are needed to elucidate the molecular details of these processes.

## Discussion

The cellular damage of mistranslation can have many different sources [1], including (i) loss of protein function, (ii) extra clean-up costs by overloading the quality-control systems involved in degradation or refolding of misfolded proteins, and (iii) induction of toxicity by protein aggregation. To investigate how organisms mitigate the deleterious effects of mistranslation during evolution, a mutant tRNA was expressed in *S*. *cerevisiae*. The construct induced over a 1,000-fold increase in mistranslation at the CUG codon and exposed the global detrimental effects of codon-specific ambiguity on the proteome. By integrating evolutionary experiments and genomic and functional analyses of the evolved lines, the following main conclusions were reached.

First, the fitness defect due to mistranslation was rapidly mitigated during laboratory evolution. Evolution reduced the rate at which errors occur (error prevention) and mitigated the harmful effects of errors (error mitigation) as well. Interestingly, we failed to find evidence for mutations at individual CTG sites. This is not completely unexpected, as mistranslation at CUG codons affects thousands of sites simultaneously, each of which probably have a relatively small contribution to fitness individually. Therefore, the selection pressure for reduction of mistranslation rate locally (i.e., at individual genic sites) may be too small to be detected in the laboratory.

Second, our work demonstrates that mistranslation initiates rapid evolution of genomic architecture (see also [41]). Convergent evolution of nearly identical chromosomal duplications indicates that these mutational events confer specific fitness advantages [42–44], most likely by simultaneous dosage increment of protein complexes and cellular subsystems involved in error mitigation. Indeed, chromosomal duplications are known to promote microbial evolution under environmental stress [45–47]. Interestingly, we found repeated occurrence of loss of function mutations in a repressor of the transcription of histone gene (*HIR2* [S000005564]). Inactivation of *HIR2* decreases heterochromatin-mediated gene silencing and increases chromosomal instability due to defective kinetochore formation [48,49]. Future work should elucidate whether *HIR2*-mediated chromosomal instability promotes the rise of adaptive duplicated chromosome segments during evolution.

Third, reduction of protein aggregation was a main target of evolution. Indeed, maintenance of proteome homeostasis is crucial for cell survival [50]. Proteins must reach their native conformation, refolded when necessary, and damaged proteins must be degraded. Folding and degradation of misfolded proteins are assisted by the concerted action of molecular chaperones and the proteasome [51]. In times of proteotoxic stress caused by mistranslation, these protein quality-control mechanisms are overwhelmed, leading to the accumulation of misfolded proteins and protein aggregates [52].

Fourth, the evolutionary adjustment of proteome homeostasis to mistranslation is achieved through acceleration of protein turnover [53], a process that is determined by the combined rates of protein synthesis and ubiquitin-proteasome system mediated degradation. This is in agreement with prior works suggesting that proteins with high turnover rate and, thus, with short lifetime will have lower risk of misfolding than long-lived proteins [30]. As newly synthesized polypeptides compete for the protein folding machinery, a large fraction of error-free proteins are degraded shortly after translation [1]. Deubiquitinating enzymes, such as Ubp3p, have a central role in rescuing misfolded but partly active proteins from proteasomal degradation [32].

Fifth, there is a strong evolutionary trade-off between survival under starvation and adaptive mechanisms underlying tolerance to mistranslation. The evolved lines showed fitness defects and impaired capacity to degrade mature ribosomes upon nutrient limitation. Moreover, as a response to the energy demands of accelerated protein turnover, the evolved lines exhibited increased glucose uptake partly achieved by selective duplication of hexose transporter genes.

To summarize, accelerated protein turnover is an effective first line of defense against protein mistranslation, but due to its exceptionally high energetic demand, this strategy is only feasible in nutrient-rich environments. This leads to the prediction that translation fidelity should vary across environments: microbes living in nutrient-poor conditions are expected to show much less tolerance to mistranslation. Due to the shortage of comparative data on translational fidelity across related microbial species, testing this prediction is not yet possible.

Robustness to mistranslation in our study was achieved by disposing of proteins prone to aggregation rather than by improving the efficiency of protein folding of individual proteins. An unexpected aspect of our work is that there were no mutations found in known chaperone-encoding genes. Moreover, functional enrichment analysis (Table 2) revealed that the accumulated mutations had no major effect on the expression level of chaperone-encoding genes. Probably, such mutations would be of little or no benefit, as a large fraction of mistranslated proteins in our study is damaged beyond repair. Indeed, CUG-encoded residues in yeast proteins are mostly buried and located in functionally conserved positions [17]. Moreover, given the large differences in Ser and Leu in chemical properties (including hydrophobicity), this amino acid change is expected to cause abrupt changes in protein structure.

Our work has several other implications for future studies. It supports the idea that partial or complete chromosome duplications fuel rapid evolutionary adaptation [42–44]. By altering the dosages of numerous, functionally related proteins simultaneously, these genetic changes introduce large phenotypic leaps that enable adaptation even in relatively small populations [44]. However, a prior study suggested that due to their deleterious side effects, chromosomal duplications are only transient solutions that are later augmented or replaced by more refined mechanisms through individual point mutations [47].

Protein turnover decreases with age in unicellular and multicellular organisms alike, resulting in an increase in the amount of intracellular damaged proteins [53]. Decreased protein turnover rate with age is the result of cumulative damage to the various components of the protein synthesis and degradation machinery [53]. Our work indicates that translational fidelity and the ubiquitin-proteasome system are functionally linked to each other and they could have a strong influence on cellular longevity.

Our work focused on the detrimental effects of mistranslation. However, as mistranslation triggers an unfolded protein stress response, it also facilitates survival under acute stress [16,54]. It would be important to see whether evolution of tolerance to mistranslation interferes with such adaptation. There might be a strong negative trade-off between cellular robustness to mistranslation and mistranslation-mediated preadaptation to stressful conditions. This possibility can readily be tested in the laboratory.

Finally, our work also sheds light on the problem of genetic code evolution [55]. Natural alterations to the standard genetic code have been discovered in many species. Interestingly, *Candida albicans* maintained variable serine and leucine incorporation levels at CUG sites [56]. The “ambiguous intermediate” theory states that tRNA mutations expand the decoding capacity of tRNA, leading to a transient state of ambiguous decoding of a single codon by both its cognate tRNA and the mutant tRNA. Proponents of the theory argued that such codon ambiguity is an important first step for gradual codon reassignments [57]. However, this initial step of ambiguous decoding is expected to lead to a serious decline in fitness due to the synthesis of non-functional or toxic proteins. Our work suggests that this problem is not fatal for the ambiguous intermediate theory. Selection pressure against genotypes with ambiguous decoding may only be temporary, as organisms can readily evolve tolerance to mistranslation.

## Materials and Methods

### Yeast Strains and Plasmids

*S*. *cerevisiae* strains used in this study were self-diploids based on BY4742 background (MATα; *his3Δ1; leu2Δ0; lys2Δ0; ura3Δ0*). CUG ambiguous cells were obtained by transformation with the single-copy *LEU2* plasmid containing the *C*. *albicans* G_33_-tRNA_CAG_^Ser^ gene (pUKC715) [18]. Control cells were transformed with the single-copy vector pRS315 (vector alone). Yeast transformations were carried out using the lithium-acetate method [58]. Transformants were selected on leucine dropout synthetic complete medium (SC-leucine: 5g/L ammonium sulfate, 1.7 g/L Yeast Nitrogen Base, supplemented with 2% glucose and with amino-acid mix, without leucine).

### Laboratory Evolution

Laboratory evolution experiment was conducted using leucine dropout synthetic complete liquid medium (SC-leucine). Starting from a single clone carrying tRNA_CAG_^Ser^, 11 populations were inoculated into 100 μl liquid medium in a 96-well format plate (Greiner) and were incubated at 30°C in a shaking incubator. Using the same protocol, we also established 11 control wild-type evolving lineages carrying the corresponding empty vector. One percent of the stationary phase cultures were transferred to fresh medium every second day using handheld pintools (VP407, V&P Scientific, Inc.). The laboratory evolution was carried out for 72 d.

### Fitness Measurements

We used established protocols specifically designed to measure fitness in yeast populations [21,22]. The fitness of the ancestor and the evolved lines were measured in 10–20 replicates each. The growth curves were monitored over a 48 h incubation period in a Biotek Powerwave XS2 automated plate reader in 384 density plates filled with SC-leucine liquid medium. During the kinetic run, the optical density of each well was recorded at 600 nm (OD_600_) every 4.5 min. Between the optical readings, the cultures were incubated at 30°C, with alternating shaking speed (1,000–1,200 rpm). Growth rate was used as proxy for fitness and was estimated as previously [59].

### Sensitivity to Protein Synthesis Inhibitors

To test the sensitivity to protein synthesis inhibitors, the culture medium was supplemented with subinhibitory concentration of cycloheximide (0.06 μg/ml, Biochemica). Fitness was estimated as above.

### Sensitivity to Nutrient Limitations

To test the sensitivity under nutrient limitations, culture medium with limited carbon source (SC-leucine supplemented with 1% glucose) and culture medium with limited amino acid source (SC-leucine supplemented with 0.25% amino acid dropout mix) was used. Fitness was estimated as above.

### Plasmid Swap

To test whether the detected fitness improvements in the evolved lines were not due to loss of function mutations in the mistranslating tRNA_CAG_^Ser^ plasmid, which could potentially arise during the course of laboratory evolution, the tRNA_CAG_^Ser^ plasmid was swapped with the original tRNA_CAG_^Ser^ plasmid in the evolved lines. To investigate the effects of genomic mutations in the absence of high mistranslation rate, the tRNA_CAG_^Ser^ carrying plasmid was eliminated, and the empty vector was introduced into the evolved lines. For each strain, we confirmed tRNA_CAG_^Ser^ loss and subsequent plasmid gain using the appropriate plasmid marker.

### Quantification of Protein Synthesis

The quantification of translation rate was done by using protein pulse labeling method with [^14^C(U)]-L-Amino Acid Mixture [25,26]. Amino acid incorporation was performed for the wild type and all evolved lines without the plasmid containing the tRNA_CAG_^Ser^ in 15 replicates each. Briefly, 2 × 10^7^ cells were collected, re-suspended into 2 ml of pre-warmed minimal medium and the suspension was incubated 20 min at 30°C with agitation. 20 μl of cold [^14^C(U)]- L-Amino Acid Mixture was added (Perkin Elmer, 0.1 mCi/ml) and the mixture was incubated 10 min at 30°C with agitation. Amino acid incorporation was stopped by the addition of 60 μl of cycloheximide (20 mg/ml) and ice incubation. Cells were washed once with cold water and frozen at -80°C. Protein was then extracted by re-suspending cell pellets in 300 μl Lysis buffer (50 mM potassium phosphate buffer pH 7, 1 mM EDTA, 5% (V/V%) glycerol, 1 mM phenylmethylsulfonyl fluoride, complete mini protease inhibitor cocktail (Roche) and 100 μl of glass beads. Cells were disrupted using a Precellys (Bertin Technologies, Montigny-le-Bretonneux, France) disrupter (five cycles of 10 s at 5,000 rpm and 1 min on ice between cycles) and centrifuged at 5,000 g for 10 min. A total of 50 μl of supernatant was applied on 1 cm^2^ square paper microfiber filter (GF/C, Whatman, Maidstone, United Kingdom). Amino acid incorporation was measured using a scintillation counter (Beckman) and protein extracts were quantified using the bicinchoninic acid (BCA) protein quantification Kit (Pierce. Rockford, IL, United States). [^14^C(U)]-L-Amino acid incorporation was normalized against the total amount of protein for each.

### Measuring Mistranslation Rate

In order to check for a possible reduction in mistranslation rate, the misreading activity of the tRNA_CAG_^Ser^ in the ancestor and evolved lines was tested using the β-galactosidase method [10]. Nine replicates of ancestor and evolved lines were used per assay. The *E*. *coli LacZ* gene contains 54 CTG codons and misincorporation of Ser at these Leu codons generates a combinatorial array of mutant β-galactosidase molecules (statistical β-gal) whose altered stability can be quantified using thermal denaturation assays.

Yeast cells containing the empty vector and the tRNA_CAG_^Ser^ plasmid, respectively, were co-transformed with the pUKC815 plasmid, which contains the promoter of yeast phosphoglycerate kinase (*PGK1* [S000000605]) gene, the N-terminal 33 amino acids of *PKG1* gene fused in frame to the *E*. *coli lacZ* gene, encoding β-galactosidase [20]. Yeast cells expressing both plasmids were selected in leucine and uracil dropout SC medium. Approximately 2.5 × 10^6^ ancestor and evolved cells from the exponential phase were harvested by centrifugation, respectively. Cells were washed and resuspended in 800 μl of Z-buffer (60 mM Na_2_HPO_4_, 40 mM NaH_2_PO_4_·2H_2_O, 10 mM KCl, 1 mM MgSO_4_·7H_2_O, 50 mM 2-mercaptoethanol, pH 7.0), 20 μl of 0.1% SDS, and 50 μl of chloroform. Cell suspensions were mixed for 30 s and incubated in triplicate at 47°C in a water bath for 10 min. This β-galactosidase unfolding step was followed by a refolding step, which was carried out by incubating samples on ice for 30 min. Residual β-galactosidase activity was then quantified at 37°C. For this, the assay tubes (200 μl) were incubated for 5 min at 37°C and then 4 mg/mL of the *ο*-nitrophenyl-β-D galactopyranoside (ONPG, Sigma-Aldrich) substrate were added to each tube. After 5 min, the reactions were stopped by the addition of 400 μl of 1M Na_2_CO_3_. β-galactosidase activity was determined by monitoring *ο*-nitrophenol synthesis at 420 nm and normalized for the total β-galactosidase produced by each line.

### Quantification of β-galactosidase Expression (Western Blot)

To control for potential differences in total β-galactosidase expression across strains, we quantified β–galactosidase expression level using western blot. Total protein fractions were analyzed under reducing conditions using 10% SDS-PAGE and blotted onto nitrocellulose membranes (Hybond ECL, Amersham) according to standard procedures. β-galactosidase was detected using anti-β-gal rabbit IgG primary antibody (Invitrogen) at 1:5,000 dilution. Bound antibody was visualized by incubating membranes with a IRDye680 goat anti-rabbit secondary antibody (Li-cor Biosciences, Lincoln, NE, US) at 1:10,000 dilution. Detection was carried out using an Odyssey Infrared Imaging system (Li-cor Biosciences). The amount of β-galactosidase was normalized to the amount of *ADH1* (S000005446) present in the total protein fraction.

### Measuring Protein Aggregation Rate

An established method was applied to estimate cellular propensity for protein aggregation [27]. It is based on the aggregation of a fluorescently tagged human protein (von-Hippel-Lindau [VHL-mCherry]). This human protein is prone to misfolding in the absence of its cofactor elongin BC, a protein absent from the *S*. *cerevisiae* genome. However, the active quality-control machinery of *S*. *cerevisiae* can prevent the aggregation of this fusion protein. It results disperse cytosolic localization of the mCherry. Upon accumulation of erroneous proteins, the control machinery becomes overloaded, and the human protein becomes aggregated, while the fluorescent tag remains functional. In this case, the red fluorescence will appear as an aggregated focus point. The expression of the VHL-mCherry was driven by a galactose inducible promoter, from a plasmid (pGAL-VHL-mCherry [CHFP]). Six replicates of ancestor and wild-type strains and 3 replicates of evolved lines were grown on leucine and uracil dropout SC medium, containing 2% raffinose as carbon source. Saturated cultures were diluted into the same selection medium, which contains 2% raffinose as carbon source and 2% galactose, to induce the expression of the fusion protein. Micrographs were taken from live cells using PerkinElmer Operetta High Content Imaging System. The ratio of cells with aggregated foci was calculated by dividing the number of cells with aggregated foci with the total number of cells expressing VHL-mCherry.

### Quantification of tRNA Expression by Northern Blot

The level of the mistranslating tRNA (expressed from single-copy plasmids) was monitored by northern blot analysis. *C*. *albicans* SN148 ancestor empty vector cells were used as positive control. Fractionation of tRNAs was carried out on 12%–15% polyacrylamide (40% Acril:Bis) gels containing 8 M urea (0.8 mm thick, 30 cm long). In each gel slot, 50 μg of total RNA sample was loaded and gels were electrophoresed at 500 V for 16 h. Fractioned tRNAs were localized by UV shadowing, the portion of the gel containing tRNAs was cut and transferred onto a nitrocellulose membrane (Hybond N, Amersham) using a Semy-dry Trans Blot (Bio-Rad). For hybridization, probes were prepared using 10 pmol of dephosphorylated oligonucleotide and 4 μl of ɣ-^32^P-ATP (5000Ci/mmol) (Perkin Elmer) in 1x T4 kinase buffer, 10 mM spermidine and 16 units of T4 kinase (Takara). Labelling reactions were incubated at 37°C for 1 h and then probes were extracted using phenol:chloroform:isoamyl alcohol (PCIA). The hybridization protocol was performed as described by Jacques Heitzler and colleagues [60]. Membranes were pre-hybridized for 30 min at 55°C in a hybridization solution [5x Denhardt’s solution (1% Ficol, 1% Polyvinylpyrrolidone and 1% Bovine serum albumin), 6x SSPE (3 M NaCl, 173 mM NaH_2_PO_4_, 25 mM EDTA) and 0.05% SDS]. Membrane hybridizations were performed overnight in the above buffer using probes GCGACACGAGCAGGGTTC for detection of tRNA^Ser^_CAG_ and GCGGAAGCCGGGAATCGAAC for detection of the control tRNA^Gly^_CCC_. Membranes were then washed four times (3 min each time) in 2x SSPE, 0.5% SDS at 53°C and exposed overnight with intensifying screens and developed using a Molecular Imager FX (Bio-Rad).

### Proteasome Activity Quantification

In ancestor and evolved lines, proteasome activity was quantified as described previously [19] in three replicates each. From the middle of the exponential phase, 2 × 10^8^ cells were collected, washed and frozen at -80°C. Cell pellets were resuspended in 350 μl of lysis buffer (10 mM Hepes, 10 mM KCl, 1.5 mM MgCl), two-thirds volume of glass beads and were disrupted using a Precellys disrupter (three cycles of 10 s at 5,000 rpm followed by 2 min on ice between cycles). Pellets were centrifuged for 5 min at 3,000 g followed by 10 min at 15,000 g. Protein extracts were quantified using the BCA protein quantification kit (Pierce). 100 μg of protein extracts were resuspended in assay buffer (10 mM Tris pH 8, 20 mM KCl, 5 mM MgCl) to a final volume of 100 μl and were incubated at 37°C for 15 min. The proteasome substrate N-SLLVY-MCA (Sigma) was added to a final concentration of 50 μM, and cells were incubated at 37°C during 60 min with agitation. Activity was measured using a Perkin Elmer Luminescence Spectrometer (LS 50B) at 365 nm (excitation) and 435 nm (emission).

### Measuring Glucose Uptake

Glucose uptake of the cells were measured as described previously [35]. Briefly, 3–3 replicates of wild type and evolved lines (in absence of the mistranslating tRNA_CAG_^Ser^) were inoculated into 20 ml of leucine dropout SC liquid medium to an initial optical density of 0.01. The cultures were incubated at 30°C, shaking at 200 rpm. Optical densities were recorded every 3 h. Glucose content of the media was measured using Glucose assay HK kit (Sigma) in parallel with optical density measurements. To assess glucose uptake rate, the measured glucose content in the medium was normalized to the cell number (calculated from the OD) of the given line.

### Quantification of the Ribosomal Protein Rpl25p

Ancestor and evolved strains expressing the tRNA_CAG_^Ser^ were transformed with plasmid pRS316-*RPL25*-GFP [38] and were grown in minimal medium without leucine and uracil to an OD_600_ = 0.2–0.8. Nine to 11 replicates were used for each strain. Cells were pelleted at 3,000 g for 5 min. The supernatant was removed and after a washing step, the cells were incubated for 24–48 h in starvation medium (0.17% yeast nitrogen base without amino acids and without ammonium sulfate, 2% glucose). Cells were then poured onto a microscope slide previously coated with a bed of 1% of agarose. The localization of GFP-tagged proteins was scored after 24 h using a Zeiss MC80 Axioplan 2 light microscope, equipped for epifluorescence microscopy with the filter set HE38. Microscope fields were randomly chosen and at least 100 cells were analyzed per sample. Photographs were taken using an AxioCamHRc camera and the number of cells with vacuolar fluorescence was counted and normalized for the total number of cells [61].

### Starvation Assay

To measure chronological lifespan, a previously described method was used [38]. Briefly, the ancestor and evolved lines (in absence of tRNA_CAG_^Ser^) were inoculated into 10 ml leucine dropout SC medium, three replicates each. The medium was supplemented with a 2-fold excess of the histidine, methionine and uracil to avoid possible artifacts due to auxotrophic deficiencies of the strains. Initial densities of the cultures were set to OD_600_ = 0.05. After 3 d of growth, when the cultures reached saturation, aliquots from the culture were collected and were plated onto YPD plates in serial dilutions. The plates were incubated in 30°C for 2–3 d, and viability was assessed by counting colony forming units. The viability at this time point was considered as initial viability (100%). Cells from saturated cultures were washed and resuspended in sterile water, to remove excess nutrients, originating from autolysed cells. The cultures were kept on incubating at 30°C with shaking, and in every 3 d, the same volume of aliquots were collected and subjected to viability assessment as mentioned above.

### Genomic Expression Profiling

Total RNA was extracted using the hot-phenol method [62] with few modifications. Briefly, 50 ml of exponentially growing cells were harvested and frozen overnight at −80°C. Cell pellets were resuspended in 0.5 ml of lysis buffer (10 mM Tris pH 7.5, 10 mM EDTA, 0.5% SDS) and 0.5 ml of acid phenol chloroform (5:1 pH 4.7, Sigma). The samples were vigorously mixed and heat incubated at 65°C for 1 h. The aqueous phase was separated from the phenolic phase by centrifugation at 4°C. The aqueous phase was re-extracted with the same volume of chloroform:isoamyl alcohol (24:1, Fluka). RNA was precipitated overnight at −80°C with ethanol 100% and 3 M sodium acetate pH 5.2. RNA was pelleted by centrifugation and resuspended in sterile MilliQ water. Total RNA samples were treated with *DNase*I (Amersham Biosciences) according to the commercial enzyme protocol and quantification and quality control was performed using the Agilent 2100 Bioanalyzer system.

Gene expression profiling was performed using the Agilent protocol for One-Color MicroarrayBased Gene Expression Analysis Quick Amp Labeling v5.7 (Agilent Technologies). Briefly, cDNA was synthesized from 600 ng of total RNA using Agilent T7 Promoter Primer and T7 RNA Polymerase Blend and labeled with Cyanine 3-CTP. Labeled cDNA was purified with RNeasy mini spin columns (QIAGEN) to remove residual Cyanine 3-CTP. Dye incorporation and quantification was monitored using the Nanodrop 1000 Spectrophotometer.

To prepare hybridization, 1.65 μg of Cy3-labeled cRNA were mixed with the fragmentation mix (Blocking Agent and Fragmentation Buffer) and incubated for 30 min at 60°C. Finally, GEx Hybridization Buffer HI-RPM was added and the preparation was assembled in the custom made Agilent arrays (yeast G4813A). Slides were prepared using Agilent gasket slides according to the manufacturer instructions. Each hybridization was carried out for 17 h at 65°C, in an Agilent hybridization oven. After washing and drying, the microarrays were scanned using the Agilent G2565AA microarray scanner (Agilent).

### Microarray Data Extraction and Analysis

Probes signal values were extracted from microarray scan data using Agilent Feature Extraction Software (Agilent). The microarray raw data was submitted to the GEO database and has been given the following accession number: GSE65718. Data were normalized using median centering of signal distribution with Biometric Research Branch BRB-Array tools v3.4.0 software. Microarray data analysis was carried out with MEV software (TM4 Microarray Software Suite) [63]. Student’s *t* test was applied to identify genes that showed statistically significant (*p* ˂ 0.01) differences in expression between control (ancestor with empty vector) and evolved lineages.

### DNA Sequence Analysis

Genomic DNA extraction was carried out using the Genomic-tip 100/G kit (Qiagen) according to the manufacturer’s protocol. Quantification and quality assessment were performed using the Picogreen fluorescence based quantification assay.

For Illumina sequencing, genomic DNA was prepared and sequenced using the manufacturer-supplied protocols and reagents, as follows. One library per sample was constructed using Illumina DNA Sample Prep standard protocol and with an insert size of 400–500 bp. Briefly, 5 μg of high molecular weight genomic DNA (gDNA) was fragmented by Covaris sonication device. Following sonication, DNA fragments were end-repaired and A-tailed. Adapters were then ligated via a 3′ thymine overhang. Finally, ligated fragments were amplified by PCR. The library was applied to an Illumina Flowcell for cluster generation. Sequencing was performed on a Genome Analyzer IIx instrument using ~150 bp paired-end reads.

### Bioinformatic Analysis

Raw sequence data, 146 bp paired end reads with expected insert size of 400–500 bp, from each sample were trimmed by removing consecutive bases on both 5′- and 3′ flanks with base quality less than 20. Trimmed reads that did not pass filtering criteria for ambiguity (N content < 5%), complexity (score ≥ 10), length (50 bases or longer), and average base quality ≥ 20 were removed using Bamtools [64].

Remaining reads were mapped to the reference genome of *Saccharomyces cerevisiae* S288C, obtained from the Saccharomyces Genome Database [65] using BWA [66]. Processing and filtering of mapped reads were done using Samtools [67]. After removal of duplicates, read pairs aligning to opposite strands, or those where predicted insert size did not match actual size, were removed. Additionally, read pairs were removed where one or both reads had low mapping quality (MQ < 20) or had less than 95% sequence identity to the reference.

Mapped reads were analyzed using Samtools to produce read pileups [67], detect single nucleotide variants and call genotypes. Small insertions and deletions (indels) were not called. Bases with low base quality or with read depth less than three or higher than twice the sample average coverage were called as unknown genotype. Sequencing data were archived in the European Nucleotide Archive under accession number PRJEB8951.

### Functional Enrichment Analysis

Funspec (acronym for "Functional Specification"), a web-based cluster interpreter for yeast [23] was used for functional enrichment analysis. The gene set of interest was uploaded to the web interface of Funspec and clustering was done using automated algorithms, based on various knowledge resources. Intersections of the input list were sought with any given functional category. A GO category was termed as enriched significantly, if the genes annotated to a particular GO term are significantly overrepresented (*p* < 0.05) in the given gene set, using the whole genome as the background set of genes.

### Accession Numbers

The accession numbers for genes mentioned in this paper are from the Saccharomyces Genome Database (http://www.yeastgenome.org).

## Supporting Information

S1 Data All data used to create figures in the manuscript. The file contains the datasets used to create the manuscript figures. Specifically, it contains relative fitnesses, genomic expression changes, protein synthesis rates, relative B-gal activities, the ratio of cells with aggregated foci, relative proteasome activities, glucose uptake rates, live cell number ratios, and Rpl25p-GFP ratios in vacuole in the evolved and ancestor lineages.
(XLSX)

S1 Fig Laboratory evolution under low mistranslation. Wild-type yeast cells (carrying no tRNA construct) were allowed to evolve in the laboratory. Growth rate was used as a proxy for fitness.
(TIF)

S2 Fig The sequence alignment of tRNA_CAG_^Ser^ genes isolated from the evolved lines. No mutations were found in the construct.
(TIF)

S3 Fig Expression of the mutant tRNA in ancestor and evolved lines. tRNA_CAG_^Ser^ expression was quantified relative to *Candida albicans* SN148 after normalization to the control tRNA expression (tRNA_CCC_^Gly^). The bars indicate mean ± 95% confidence interval. Two-sample *t* test was used to assess difference in fitness between ancestor and evolved lines. ** indicates *p*-value < 0.01.
(TIF)

S4 Fig Relative fitness of wild type and evolved lines under nutrient limitation. (A) Relative fitness of ancestor and evolved lines on limited carbon source (SC-leucine supplemented with 1% glucose). Growth rate (calculated by monitoring optical density) was used as a proxy for fitness. Fitness values were normalized to the wild-type control carrying no tRNA_CAG_^Ser^ construct. The bars indicate mean ± 95% confidence interval. Mann-Whitney U test was used to assess difference in fitness between ancestor and evolved lines. */**/*** indicates *p*-value < 0.05/0.01/0.001, respectively. To ensure that differences in fitness values reflect the impact of the accumulated mutations in the evolved lines (rather than the direct effects of mistranslation), tRNA_CAG_^Ser^ was swapped for the corresponding empty vector in the ancestor and the evolved lines. (B) Relative fitness of ancestor and evolved lines on limited amino acid source (SC-leucine supplemented with 0.25% amino acid dropout mix). Growth rate (calculated by monitoring optical density) was used as a proxy for fitness. Fitness values were normalized to the wild-type control carrying no tRNA_CAG_^Ser^ construct. The bars indicate mean ± 95% confidence interval. Mann-Whitney U test was used to assess difference in fitness between ancestor and evolved lines. */**/*** indicates *p*-value < 0.05/0.01/0.001, respectively. To ensure fitness values reflect the impact of the accumulated mutations in the evolved lines (rather than the direct effects of mistranslation), tRNA_CAG_^Ser^ was swapped for the corresponding empty vector in the ancestor and the evolved lines.
(TIF)

S1 Table The list of mutated genes with non-synonymous mutations. (XLSX)

S2 Table Mutations identified by Illumina next-generation sequencing. The table contains all the identified de novo mutations in the sequenced genomes. Legend for the sheet "de novo SNPs," column name and variable description: **evolved line—**coding number of the evolved line; **chromosome—**number of the chromosome of the mutational event; **position—**position of the mutational event on the given chromosome; **reference**—nucleotide sequence of the reference strain at the given chromosomal position; **altered**—mutated nucleotide sequence at the given chromosomal position; **target gene ID**—systematic name of the gene affected by the given mutational event, or "no gene around" if the mutation is farther than 500 nt upstream, or 150 nt downstream of any gene; **bio type**—describes whether gene of "target gene ID" codes for protein or tRNA; **effect**—describes the effect of the mutational event on protein coding; **old AA/new AA**—the amino acid coding is given at the corresponding position before and after the mutational event. Legend for the sheets "de novo duplications" and "ancestral duplications," column name and variable description: **evolved line—**coding number of the evolved line; **chromosome**—number of the chromosome of the mutational event; **start position**—start position of the duplication on the given chromosome; **end position**—end position of the duplication on the given chromosome
(XLSX)

S3 Table RNA microarray analysis results. The table contains microarray data on all ancestor and evolved lines subjected to microarray analysis. Legend for the sheets "Evolved line 1" and “Evolved line 4”: **GENE SYMBOL—**The systematic name of the gene of which expression level is shown in the table in the given row; **Description**—Functional description of the gene of which expression level is shown in the table in the given row; **Log Fold change**—log2 mRNA expressions of the given evolved lineage normalized to the ancestor; **CNV (-1 chromosome deletion, +1 chromosome duplication, 0 no change)**—Copy number changes of the chromosomal region in which the give gene is located.
(XLSX)

S4 Table SNPs found in tS(AGA)D3, a serine-coding tRNA. (XLSX)

## Abbreviations

BCA: bicinchoninic acid
GFP: green fluorescent protein
GO: gene ontology
Leu: leucine residue
OD_600_: optical density at 600 nm wavelength
rRNA: ribosomal RNA
SC: synthetic complete
Ser: serine residue
SerRS: Seryl-tRNA synthetase
SNP: single nucleotide polymorphism
tRNA: transfer RNA
Ty element: transposon of yeast element
VHL: von Hippel–Lindau
β-gal: β-galactosidase
