# Supplementary figures and images for: Evolution of Robustness to Protein Mistranslation by Accelerated Protein Turnover
Source: PLoS Biol. 2015 Nov 6;13(11):e1002291. doi: 10.1371/journal.pbio.1002291 (PMC4636289; doi:10.1371/journal.pbio.1002291)

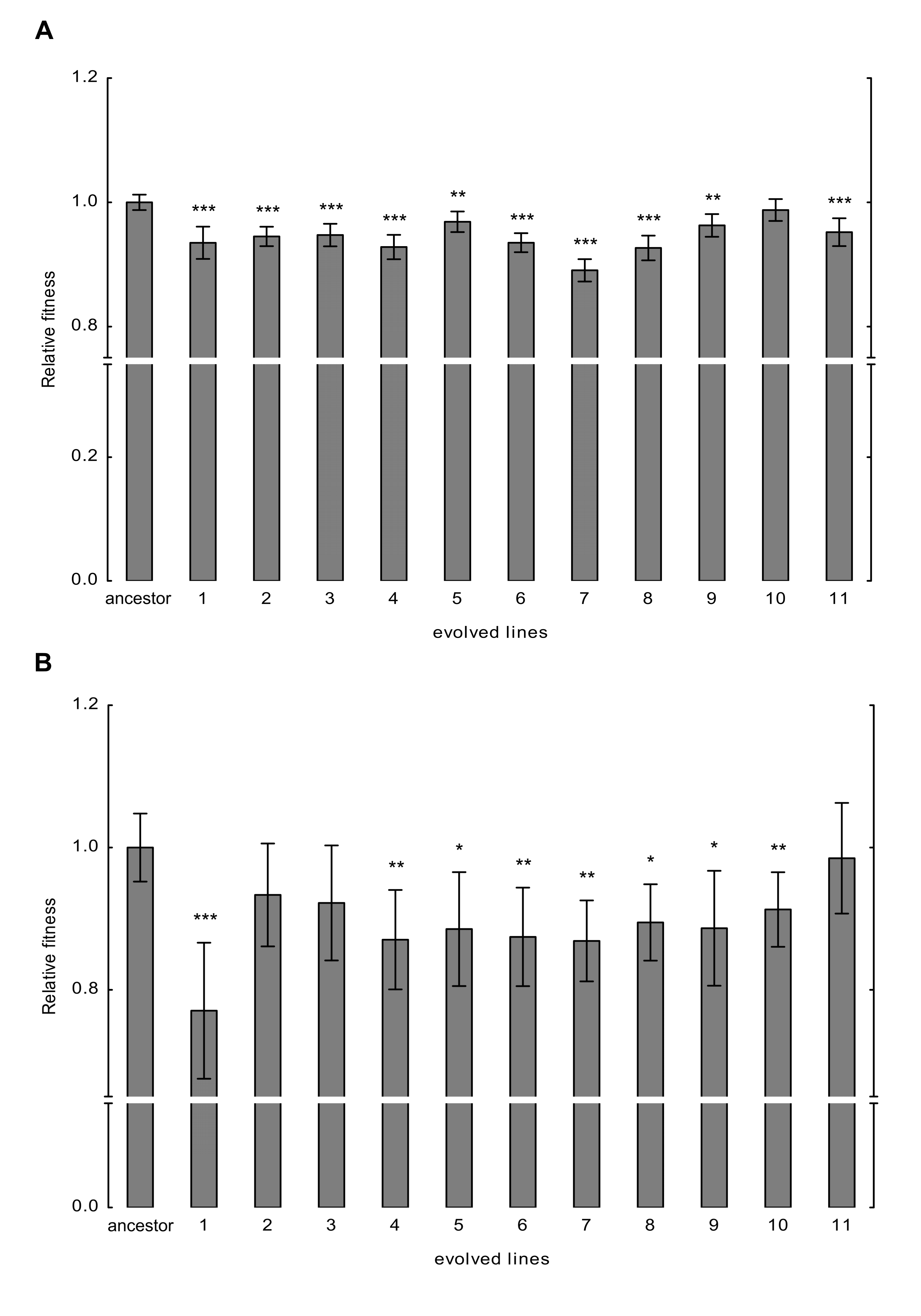

Supplement: S4 Fig — (A) Relative fitness of ancestor and evolved lines on limited carbon source (SC-leucine supplemented with 1% glucose). Growth rate (calculated by monitoring optical density) was used as a proxy for fitness. Fitness values were normalized to the wild-type control carrying no tRNACAG Ser construct. The bars indicate mean ± 95% confidence interval. Mann-Whitney U test was used to assess difference in fitness between ancestor and evolved lines. */**/*** indicates p-value < 0.05/0.01/0.001, respectively. To ensure that differences in fitness values reflect the impact of the accumulated mutations in the evolved lines (rather than the direct effects of mistranslation), tRNACAG Ser was swapped for the corresponding empty vector in the ancestor and the evolved lines. (B) Relative fitness of ancestor and evolved lines on limited amino acid source (SC-leucine supplemented with 0.25% amino acid dropout mix). Growth rate (calculated by monitoring optical density) was used as a proxy for fitness. Fitness values were normalized to the wild-type control carrying no tRNACAG Ser construct. The bars indicate mean ± 95% confidence interval. Mann-Whitney U test was used to assess difference in fitness between ancestor and evolved lines. */**/*** indicates p-value < 0.05/0.01/0.001, respectively. To ensure fitness values reflect the impact of the accumulated mutations in the evolved lines (rather than the direct effects of mistranslation), tRNACAG Ser was swapped for the corresponding empty vector in the ancestor and the evolved lines. (TIF) [file pbio.1002291.s005.tif]
